# Mosquito co-infection with Zika and chikungunya virus allows simultaneous transmission without affecting vector competence of *Aedes aegypti*

**DOI:** 10.1371/journal.pntd.0005654

**Published:** 2017-06-01

**Authors:** Giel P. Göertz, Chantal B. F. Vogels, Corinne Geertsema, Constantianus J. M. Koenraadt, Gorben P. Pijlman

**Affiliations:** 1 Laboratory of Virology, Wageningen University & Research, Wageningen, The Netherlands; 2 Laboratory of Entomology, Wageningen University & Research, Wageningen, The Netherlands; The Pennsylvania State University, UNITED STATES

## Abstract

**Background:**

Zika virus (ZIKV) and chikungunya virus (CHIKV) are highly pathogenic arthropod-borne viruses that are currently a serious health burden in the Americas, and elsewhere in the world. ZIKV and CHIKV co-circulate in the same geographical regions and are mainly transmitted by *Aedes aegypti* mosquitoes. There is a growing number of case reports of ZIKV and CHIKV co-infections in humans, but it is uncertain whether co-infection occurs via single or multiple mosquito bites. Here we investigate the potential of *Ae*. *aegypti* mosquitoes to transmit both ZIKV and CHIKV in one bite, and we assess the consequences of co-infection on vector competence.

**Methodology/Principal findings:**

First, growth curves indicated that co-infection with CHIKV negatively affects ZIKV production in mammalian, but not in mosquito cells. Next, *Ae*. *aegypti* mosquitoes were infected with ZIKV, CHIKV, or co-infected via an infectious blood meal or intrathoracic injections. Infection and transmission rates, as well as viral titers of positive mosquitoes, were determined at 14 days after blood meal or 7 days after injection. Saliva and bodies of (co-)infected mosquitoes were scored concurrently for the presence of ZIKV and/or CHIKV using a dual-colour immunofluorescence assay. The results show that orally exposed *Ae*. *aegypti* mosquitoes are highly competent, with transmission rates of up to 73% for ZIKV, 21% for CHIKV, and 12% of mosquitoes transmitting both viruses in one bite. However, simultaneous oral exposure to both viruses did not change infection and transmission rates compared to exposure to a single virus. Intrathoracic injections indicate that the selected strain of *Ae*. *aegypti* has a strong salivary gland barrier for CHIKV, but a less profound barrier for ZIKV.

**Conclusions/Significance:**

This study shows that *Ae*. *aegypti* can transmit both ZIKV and CHIKV via a single bite. Furthermore, co-infection of ZIKV and CHIKV does not influence the vector competence of *Ae*. *aegypti*.

## Author summary

Zika virus (ZIKV) and chikungunya virus (CHIKV) are highly pathogenic arthropod-borne viruses that present a serious health threat to humans. Since 2015, both viruses circulate in the same geographical regions of the Americas and are predominantly transmitted by the Yellow Fever mosquito *Ae*. *aegypti*. There is a growing number of case reports of ZIKV and CHIKV co-infections in humans, but it is uncertain whether co-infection occurred via single or multiple mosquito bites. Therefore, we infected *Ae*. *aegypti* mosquitoes via an infectious blood meal with ZIKV, CHIKV, or both and scored the saliva of (co-)infected mosquitoes 14 days post infection for the presence of ZIKV, CHIKV or both. *Ae*. *aegypti* was competent for both viruses with transmission rates up to 73% (ZIKV) and 21% (CHIKV). A substantial proportion of mosquitoes became saliva-positive for both viruses (12%), suggesting that *Ae*. *aegypti* can transmit both CHIKV and ZIKV via a single bite. Additionally, co-infections did not influence the infection or transmission rates of either ZIKV or CHIKV. Our results indicate that co-infection of CHIKV and ZIKV can lead to simultaneous transmission by the same mosquito in the field.

## Introduction

Zika virus (ZIKV; family *Flaviviridae*, genus *Flavivirus*) is a pathogenic arthropod-borne (arbo)virus that causes neurological disease in humans and congenital syndrome in newborns and infants [1]. In the 60 years after its discovery in 1947, sporadic ZIKV infections were reported in African countries and in parts of Asia [2]. The first larger ZIKV virus outbreak was reported in 2007 on the Yap Islands of Micronesia after which the virus quickly spread to other countries in south-east Asia, such as French Polynesia in 2013, and Cook Islands and Easter Island in 2014 [3]. In 2015, there was a dramatic increase of reported ZIKV cases in South America, especially Brazil where over 200,000 cases of infection, six deaths and over 2,200 incidents of ZIKV associated congenital syndrome were reported [4]. Prior to the ZIKV outbreak in the Americas, flavivirus infections linked to congenital disease were rarely reported. However, a causal relationship between ZIKV infection in pregnant women and subsequent birth malformations, such as microcephaly, has now been confirmed [1,4,5]. The main vector for ZIKV transmission is the *Aedes aegypti* mosquito [6–9], while *Ae*. *albopictus* [9–11], *Ae*. *vittatus* [12] *Ae*. *luteocephalus* [12], and *Ae*. *hensilli* [13] can transmit ZIKV in laboratory studies.

Other mosquito-borne viruses that circulate concurrently with ZIKV in South America include chikungunya virus (CHIKV; family *Togaviridae*, genus *Alphavirus*), dengue virus (DENV; family *Flaviviridae*, genus *Flavivirus*) and yellow fever virus (family *Flaviviridae*, genus *Flavivirus*). In 2013, CHIKV was introduced into South America via the Caribbean. Since then over 319,000 cases of infection and 135 deaths have been reported in South America [14]. CHIKV strains that circulate in the Americas are predominantly transmitted by *Ae*. *aegypti* mosquitoes [15]. Since CHIKV and ZIKV co-circulate in the same geographical regions, individuals can become co-infected with both viruses [16,17]. Co-infections of patients with ZIKV and CHIKV already occurred in South America [18–20], some even reporting triple infection with ZIKV, CHIKV, and DENV [21–23]. Whether a single bite of *Ae*. *aegypti* can transmit both ZIKV and CHIKV simultaneously, or whether sequential bites of two infected mosquitoes are required for such co-infections in humans, remains unclear.

Here we designed a dual-colour immunofluorescence assay that can concurrently detect ZIKV and CHIKV infection in mammalian and mosquito cells. We analysed the effect of co-infections on virus growth kinetics in mammalian and mosquito cell lines. Furthermore, we studied the effect of co-infection with both ZIKV and CHIKV on the infection and transmission rates of both viruses in *Ae*. *aegypti* mosquitoes. Finally, mosquito transmission rates after an infectious bloodmeal and intrathoracic injections were compared to study the effects of the midgut and salivary gland barriers on co- and single-infections.

## Materials and methods

### Cell culture

African green monkey kidney Vero E6 (ATCC CRL-1586) cells were cultured in Dulbecco’s modified Eagle medium (DMEM; Gibco, Carlsbad, CA, United States) containing 10% fetal bovine serum (FBS; Gibco), penicillin (100 U/ml; Sigma-Aldrich, Saint Louis, MO, United States), and streptomycin (100 μg/ml; Sigma-Aldrich) (P/S). Vero cells were cultured as monolayers in T25 cell culture flasks (Greiner Bio-One, Kremsmünster, Austria) at 37°C with 5% CO_2_, and split every 3–4 days. Prior to infections, Vero cells were seeded in DMEM containing 4-(2-hydroxyethyl)-1-piperazineethanesulfonic acid (DMEM-HEPES; Gibco) supplemented with 10% FBS, penicillin (100 U/ml), and streptomycin (100 μg/ml), hereafter named DMEM-supplemented. *Aedes albopictus* C6/36 cells (ATCC CRL-1660) were cultured in Leibovitz L-15 medium (Gibco) supplemented with 10% FBS, 2% tryptose phosphate broth (Gibco), and 1% nonessential amino acids (Gibco), hereafter named Leibovitz-complete. *Aedes aegypti* Aag2 cells were cultured in Schneider’s *Drosophila* medium (Lonza, Basel, Switzerland) supplemented with 10% FBS, hereafter named Schneider’s-complete. Both C6/36 and Aag2 cells were cultured as monolayers in T25 flasks at 28°C, and split every 3–4 days.

### Virus stocks

All proceedings involving infectious virus were executed in the biosafety level 3 laboratory at Wageningen University & Research. An infectious clone derived chikungunya virus 37997 strain (CHIKV^37997^) was used in all studies. To prepare the chikungunya virus 37997 infectious clone (pCHIK_IC_-37997), the 37997 structural cassette was produced synthetically with AscI/EcoRI overhangs (Baseclear, Leiden, The Netherlands) and cloned into the previously described CHIKV 37997 replicon CHIKrep-FlucEGFP to replace the Fluc-EGFP fusion gene [24]. CHIKV 37997 RNA was *in vitro* transcribed from 5 μg PacI (New England Biolabs (NEB), Ipswich, MA, United States) linearized pCHIK_IC_-37997 using SP6 RNA polymerase (NEB) following the manufacturer’s protocol. Vero cells were seeded one day prior to infection in 6 well cell culture plates (Greiner Bio-One) until a confluency of ~80% was reached. The culture medium was replaced for Opti-Mem (Gibco) and 3 μl of *in vitro* transcribed RNA was transfected into Vero cells using 2.5 μl Lipofectamine 2000 (Invitrogen, Carlsbad, United States). Four days post transfection the cell culture medium was harvested, centrifuged and stored at -80°C until further use (P0). In total, 500 μl P0 was used to inoculate a T75 flask (Greiner Bio-One) of C6/36 cells. Four days post infection (dpi) the cell culture medium was harvested (P1), centrifuged and the supernatant was stored in aliquots at -80°C. Virus titers were determined by end point dilution assay (EPDA) on Vero cells.

Zika virus Suriname strain 011V-01621 (ZIKV^SUR^ GenBank accession number, KU937936) [5], was obtained through the European Virus Archive Goes Global catalogue (www.european-virus-archive.com/virus/zika-virus-strain-suriname-2016) as a P3 stock grown on Vero cells. ZIKV P4 was generated by inoculating a pre-seeded T75 flask of Vero cells with 250 μl ZIKV_SUR_ P3. The supernatant was harvested (P4) at 2 dpi, centrifuged to remove cell debris, and the supernatant was stored in aliquots at -80°C. Virus titers were determined by EPDA on Vero cells.

### Virus titrations

Vero cell suspensions were retrieved by detaching Vero cells from a T25 flask with 1 ml of Trypsin-EDTA (Gibco), after which 4 ml of DMEM-supplemented was added. Virus stocks were thawed, vortexed and serial dilutions were made in DMEM-supplemented. Vero cell suspensions were diluted 1:4 with DMEM-supplemented and added to the virus dilutions in a 1:1 ratio. 10 μl of the inoculated dilutions was plated in 6-fold in micro-titer plates (Nunc, Roskilde, Denmark). EPDAs of samples infected with one virus were scored at 3 dpi based on virus induced cytopathic effect (CPE). EPDAs of co-infected samples were fixed with 4% paraformaldehyde and scored by immunofluorescence assay (IFA) at 3 dpi.

### Virus growth curves

Cell monolayers were seeded in 6-well plates and infected on the same day for C6/36 and Aag2 cells, or the next day for Vero cells. The cell culture fluid was removed and infections were performed at an MOI of 0.1 (5.7 × 10^4^–2.2 × 10^5^ TCID_50_) in standard culture media in a total volume of 1 ml. After 1 h the inoculum was removed and the monolayers were washed twice with 1 ml of Phosphate Buffered Saline (PBS), before addition of 2 ml fresh culture medium. C6/36 and Aag2 cells were maintained at 28°C and Vero cells were maintained at 37°C and 5% CO_2_. Samples of 100 μl were taken at 0, 24, 48, 72 and 96 hours post infection (hpi) and stored at -80°C until titration by EPDA on Vero cells.

### Dual-colour immunofluorescence assays

Cells were fixed with 4% paraformaldehyde/PBS for 1–3 h. Monolayers were washed 3x with PBS, permeabilized by 10 min incubation in 0.1% SDS in PBS, and washed 3x with PBS. Monolayers were stained with α-CHIKV-E2 (Rabbit Polyclonal; 1:5000; [25]) and pan-Flavivirus α-E (4G2; Mouse monoclonal; 1:50 [26]) in a 5% FBS solution dissolved in PBS for 1 h at room temperature (RT). Cells were washed 3x with PBS and stained with goat-α-mouse-Alexa Fluor 568 (1:2000; Invitrogen) and goat-α-rabbit-Alexa Fluor 488 (1:2000; Invitrogen) for 1 h at 37°C. Monolayers were washed 3X with PBS and visualized using an Axio Observer Z1m inverted microscope (Zeiss, Jena, Germany) in combination with an X-Cite 120 series lamp.

### Cell viability assay

Vero cell monolayers were seeded in 96-wells plates one day prior to infection and infected at an MOI of 0.1. At the indicated time-point, the medium was removed and replaced with 100 μl of passive lysis buffer (Promega, Madison, Wisconsin, USA). Cells were lysed by 10 min incubation at RT and lysates were stored at -20°C until further use. Twenty-five μl of reconstituted CellTiter-Glo Reagent (Promega) was added to 25 μl cell lysate and incubated at RT in the dark for 10 min before measuring the luminescence using a FLUOstar OPTIMA microplate reader (BMG Labtech, Ortenberg, Germany). Cell viability was calculated by normalizing the average luminescence of the sample to the averaged luminescence of the mock.

### Mosquito rearing

In all experiments female *Aedes aegypti* mosquitoes (Rockefeller strain, obtained from Bayer AG, Monheim, Germany) were used. Larvae and adults were maintained at 27±1°C with 12:12 light:dark cycle and 70% relative humidity. Adult mosquitoes were provided with 6% *ad libitum* glucose solution. Human blood (Sanquin Blood Supply Foundation, Nijmegen, The Netherlands) was provided through Parafilm using the Hemotek PS5 feeder (Discovery Workshops, Lancashire, United Kingdom). Female mosquitoes were kept together with males for 3 to 6 days in Bugdorm-1 insect rearing cages (30 x 30 x 30 cm, Bugdorm, Taiwan, China), before females were transferred to buckets (diameter: 12.2 cm, height: 12.2 cm; Jokey, Wipperfürth, Germany) and transported to the Biological Safety Level 3 facility for virus infection assays.

### Infectious blood meal

One day before blood feeding, the glucose solution was replaced by water in order to stimulate blood feeding of *Ae*. *aegypti* females. Virus solutions were made by diluting the virus to the indicated titer in DMEM-supplemented for ZIKV, and Leibovitz-complete for CHIKV. Since CHIKV was grown on C6/36 cells and ZIKV on Vero cells, we compensated for differences in cell culture media by mixing 250 μl of virus solution with 250 μl conditioned media from cultured C6/36 cells for ZIKV and 250 μl conditioned media from cultured Vero cells for CHIKV, after which 500 μl human blood was added. The infectious blood meal was offered through Parafilm using the Hemotek PS5 feeder. Mosquitoes were allowed to feed for 1 h *ad libitum* in light conditions, at 24°C and 70% relative humidity (RH). Mosquitoes were anesthetized with 100% CO_2_, placed on a CO_2_ pad and fully engorged females were selected. Immediately after selection, a selection of mosquitoes was frozen at -80°C to determine the amount of virus ingested by the mosquitoes. Exposed mosquitoes were maintained at 28°C. The glucose solution was refreshed every 2–3 days until 14 dpi.

### Intrathoracic injections

Virus dilutions of 4 × 10^7^ TCID_50_/ml were prepared by diluting the ZIKV and CHIKV virus stocks 1:1 with conditioned media taken from cultured C6/36 and Vero cells, respectively, to compensate for differences in growth media. *Ae*. *aegypti* mosquitoes were anesthetized with 100% CO_2_ and placed on a CO_2_ pad. Female mosquitoes were selected and injected with 69 nl of the prepared virus stock using a Drummond Nanoject II Auto-Nanoliter Injector (Drummond Scientific, Broomall, PA, United States). Infected mosquitoes were maintained at 28°C. The glucose solution was refreshed every 2–3 days until 7 dpi.

### Salivation assay

Fourteen days post blood meal or 7 days post injection mosquitoes were anesthetized with 100% CO_2_, and placed on a CO_2_ pad. Mosquitoes that died within the 7 or 14 days incubation period were discarded. Mosquitoes were immobilized by removing their legs and wings with forceps. The proboscis of each mosquito was inserted into a 200 μl yellow pipet tip (Greiner Bio-One) containing 5 μl of a 1:1 solution of 50% glucose solution and FBS, for a minimum of 45 min. After salivation, the mosquito bodies were added to 1.5 ml Safe-Seal micro tubes (Sarstedt, Nümbrecht, Germany) containing 0.5 mm zirconium beads (Next Advance, Averill Park, NY, United States). Each saliva sample was added to a 1.5 ml micro tube (Sarstedt) containing 55 μl DMEM-supplemented with additional Fungizone (50 μg/ml; Invitrogen), and Gentamycin (50 μg/ml; Life technologies), hereafter named DMEM-complete. Mosquito bodies and saliva samples were stored at -80°C until further processing.

### Infectivity assays

Mosquito bodies were taken from the -80°C freezer and immediately homogenized for 2 min at max speed in a Bullet Blender Storm (Next Advance). Homogenized bodies were centrifuged briefly and resuspended in 100 μl DMEM-complete medium. The homogenate was blended again for 2 min at max speed using the Bullet Blender and centrifuged for 1 min at 14.500 rpm. Mosquito saliva samples were thawed at RT. Of each body homogenate or saliva sample, 30 μl was used to inoculate a Vero cell monolayer in a 96 wells plate. After 2–3 h the inoculum was removed and replaced with 100 μl DMEM-complete. For mosquitoes infected with a single virus, the wells were scored for virus induced CPE at 3 dpi. This method was validated by comparing results based on CPE with IFA for the first replicate of mosquitoes, which gave identical results. For mosquitoes that were infected with both viruses, the supernatant was removed and monolayers were fixed with 4% paraformaldehyde in PBS at 3 dpi after which the wells were scored by dual-colour IFA. Bodies and saliva samples of a selection of mosquitoes with a fully disseminated infection of ZIKV, CHIKV, or both, were titrated by EPDA.

### Statistics

Kruskal-Wallis tests were used to test for differences between engorged viral titers, and final titers of mosquito bodies and saliva samples. If the outcome of a Kruskal-Wallis test was significant, differences among groups were further tested with Dunn’s tests and corrected with the Bonferroni correction for multiple comparisons. Infection and transmission rates were calculated, respectively, by dividing the number of female mosquitoes with infected bodies or with infected saliva by the total number of female mosquitoes in the respective treatment. Mosquitoes with infectious saliva, but uninfected body (<1%), were excluded from the analysis. Differences in infection and transmission rates were tested with Chi-squared tests. Multiple comparisons were corrected with the Bonferroni correction. All statistical analyses were done with the statistical software package R [27]. Power analysis was performed to confirm adequate sample sizes for the vector competence studies using G*Power software (Düsseldorf, Germany).

## Results

### ZIKV and CHIKV can co-infect and co-replicate in mammalian and mosquito cells

**Fig 1:**
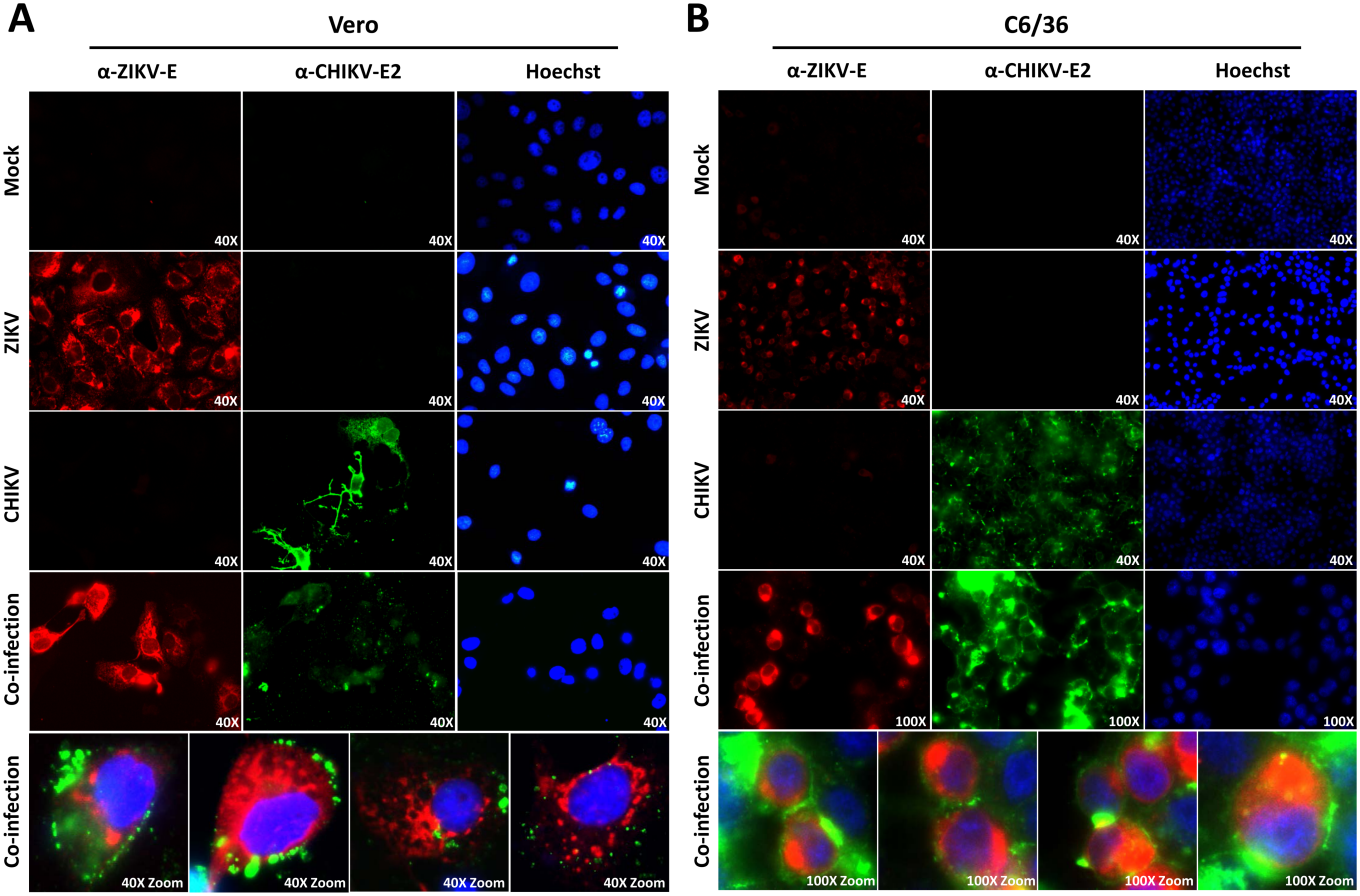
Immunofluorescence of ZIKV and CHIKV single- and co-infections in mammalian and mosquito cells. (A) Vero and (B) C6/36 cells were infected with ZIKV^SUR^, CHIKV^37997^ or both. At 48 hpi the monolayers were fixed, permeabilized, stained with antibodies for ZIKV-E (pan-Flavivirus α-E (4G2)), CHIKV-E2 and Hoechst33258 (details in Materials and methods) and visualized by immunofluorescence. Magnifications are indicated in each picture. Bottom panels indicate zoomed and merged images of co-infected cells.

A dual-colour immunofluorescence detection assay was developed to investigate whether ZIKV and CHIKV can infect and replicate in the same mammalian or mosquito cell. Vero (green monkey kidney, mammalian) cells and C6/36 (*Ae*. *albopictus*, mosquito) cells were seeded as monolayers and inoculated with ZIKV, CHIKV, or co-inoculated and stained for viral antigens at 48 hpi (Fig 1). Distinctions between ZIKV and CHIKV infected cells were clear after immunostaining in both Vero and C6/36 cells, indicating that the dual-colour immunofluorescence assay can be used to score co-infected samples for the presence of ZIKV, CHIKV, or both (Fig 1A & 1B). ZIKV infected Vero cells displayed localization of the envelope (E (pan-Flavivirus α-E (4G2))) protein predominantly near the perinuclear regions / endoplasmic reticulum, corresponding to the replication and assembly sites of flaviviruses [28] (Fig 1A). Most Vero cells in the CHIKV infected sample already lysed due to the strong CPE of CHIKV infection, as indicated by the presence of the E2 envelope protein on the remaining cell projections. Viable CHIKV infected cells showed localization of E2 near the cell boundaries, related to the assembly sites of CHIKV [29] (Fig 1A). The localization of E and E2 was similar in C6/36 cells, with ZIKV-E mostly present near the endoplasmic reticulum surrounding the nucleus, and CHIKV-E2 near the cell membrane (Fig 1B). Co-infection did not alter the localization of ZIKV-E nor CHIKV-E2, indicating that these viruses can co-infect the same cell without obvious interference. These results show that ZIKV and CHIKV are intrinsically capable to co-infect and co-replicate in cells of their mammalian and insect host.

### Growth kinetics of ZIKV and CHIKV during single- and co-infections of mammalian and mosquito cell lines

**Fig 2:**
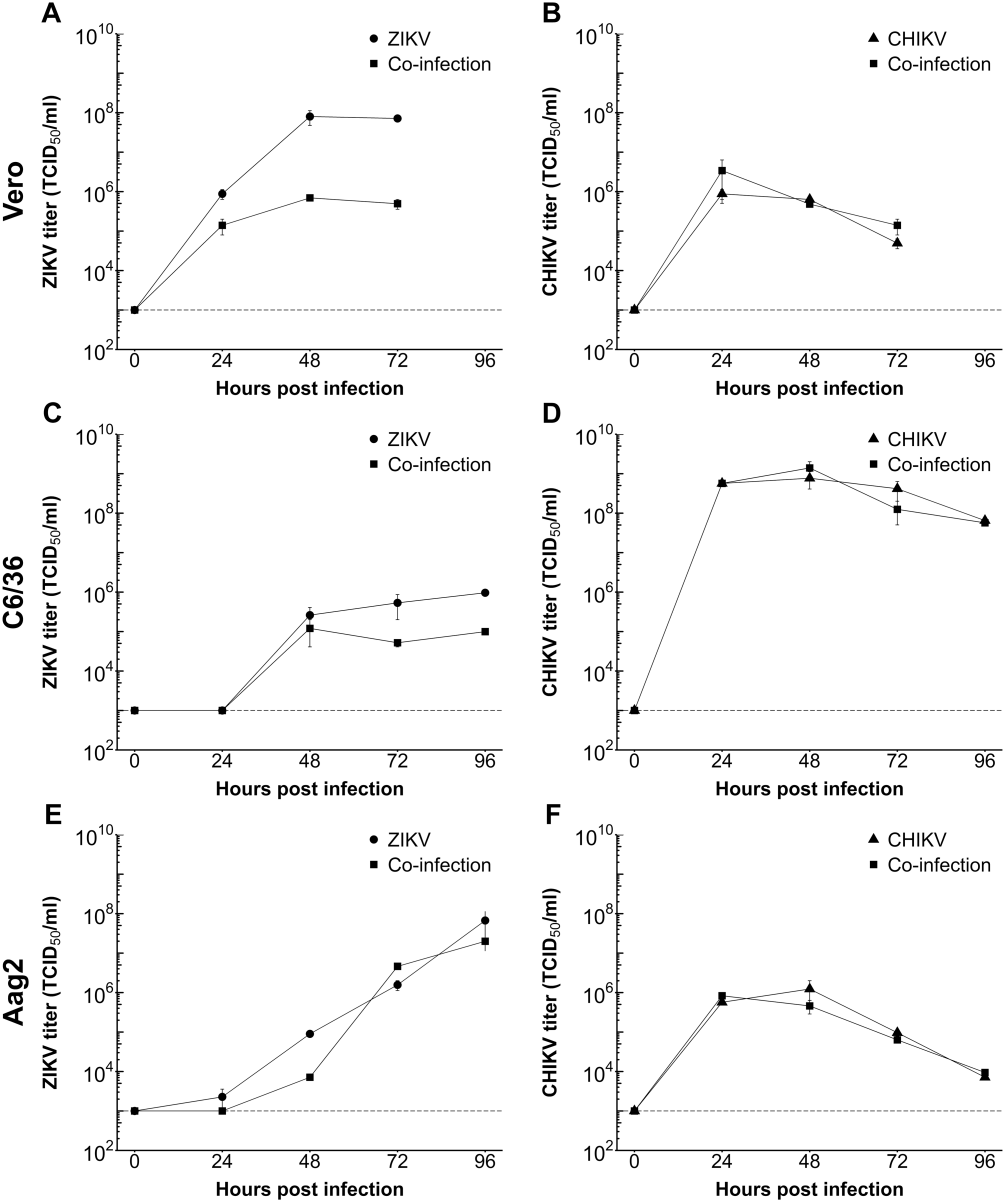
Growth curves of ZIKV and CHIKV during single- and co-infections. (A-B) Vero, (C-D) C6/36 and (E-F) Aag2 cells were infected with ZIKV^SUR^, CHIKV^37997^ or co-infected at an MOI of 0.1. The virus titers during single- and co-infection were determined by EPDA at the indicated time points for (A,C,E) ZIKV and (B,D,F) CHIKV. Shown are the mean virus titers ± SEM from duplicate samples. The detection limit of the EPDA is indicated by a dashed line.

Viral co-infections can influence the replication rate in mosquito cell lines and may affect transmission *in vivo* by the mosquito vector [30]. In order to assess whether ZIKV and CHIKV interfere with each other’s replication, the growth kinetics of ZIKV and CHIKV were determined during co- and single-infections in mammalian Vero, *Ae*. *albopictus* C6/36, and *Ae*. *aegypti* Aag2 cells (Fig 2). Cells were infected at an MOI of 0.1, washed, and culture fluid samples were collected at 0, 24, 48, 72, and 96 hpi, and titrated by EPDA. In Vero cells, ZIKV reached a peak titer of 8.0 × 10^7^ TCID_50_/ml within 48 hpi (Fig 2A), whereas CHIKV only reached a titer of 8.7 × 10^5^ TCID_50_/ml at 24 hpi (Fig 2B). During co-infection in Vero cells, the titer of ZIKV was approximately 3 logs lower at 48 hpi and 72 hpi as compared to single-infection, whereas the titer of CHIKV was not affected by co-infection with ZIKV.

In C6/36 cells, ZIKV reached a relatively low peak titer of 9.6 × 10^5^ TCID_50_/ml at 96 hpi (Fig 2C), whereas CHIKV reached peak titers of 7.7 × 10^8^ TCID_50_/ml at 48 hpi (Fig 2D). Co-infection resulted in approximately 1 log lower titer of ZIKV at 72 and 96 hpi as compared to single-infection, whereas CHIKV replication was not seemingly affected by co-infection. In Aag2 cells, ZIKV reached a peak titer of 6.7 × 10^7^ TCID_50_/ml at 96 hpi (Fig 2E), indicating that ZIKV replicates better in *Ae*. *aegypti* as compared to *Ae*. *albopictus* cells (compare Fig 2C with 2E). In contrast, CHIKV reached peak titers of 1.2 × 10^6^ TCID_50_/ml at 48 hpi in Aag2 cells, and CHIKV titers rapidly decreased at later time points (Fig 2F). This suggests that CHIKV replicates better in *Ae*. *albopictus* than *Ae*. *aegypti* cells (compare Fig 2D with 2F). Importantly, co-infections in Aag2 cells did not significantly affect the replication of either ZIKV or CHIKV (Fig 2E & 2F).

**Fig 3:**
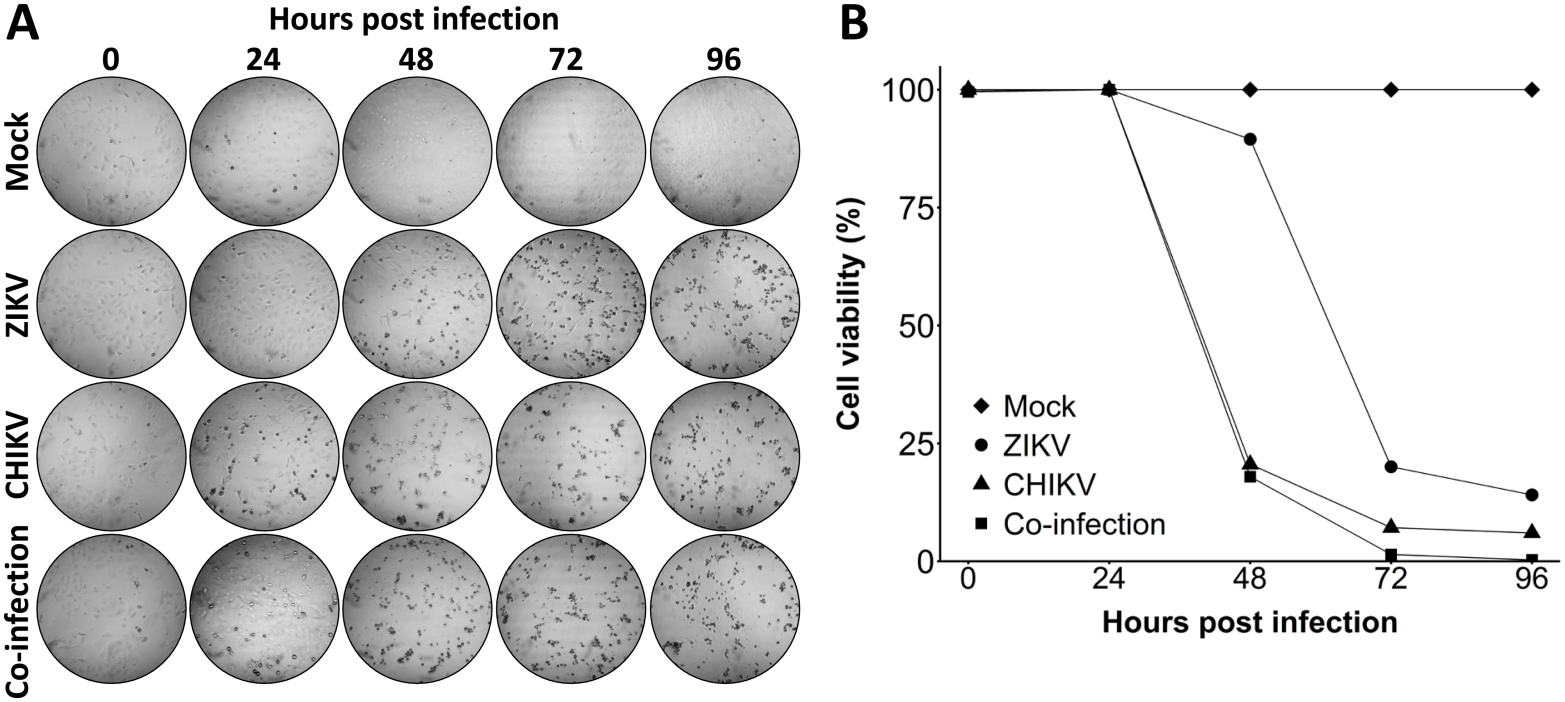
Cell viability of Vero cells during ZIKV, CHIKV single- or co-infections. (A-B) Vero cells were infected with ZIKV^SUR^, CHIKV^37997^, or co-infected at an MOI of 0.1. At 0, 24, 48, 72 and 96 hpi cells were visualized by (A) bright field microscopy and (B) lysed before measuring the cell viability by CellTiter-Glo assay. Cell viability is presented as percentage normalized to the mock.

To investigate whether the observed difference in growth kinetics of ZIKV during co- and single-infections in Vero cells was due to altered cell viability, a cell viability assay was performed (Fig 3). Signs of virus induced cytopathic effect were readily observed by bright field microscopy at 24 hpi in the CHIKV and co-infected cells, whereas ZIKV induced cytopathic effects were only observed after 48 hours (Fig 3A). Additionally, the cell viability of CHIKV infected and co-infected Vero cells was decreased dramatically to 20% at 48 hpi, whereas ZIKV maintained high cell viability up to 48 hpi (Fig 3B). These results suggest that the reduction of ZIKV titers during co-infection with CHIKV is due to the rapid and extensive CPE resulting from CHIKV-induced host-shut-off [31], which interferes with ZIKV virion production. Moreover, co-infections did not affect the cell viability in both C6/36 and Aag2 cells until 96 hpi (Cell viability: 80–100%).

### *Ae*. *aegypti* is competent to transmit ZIKV and CHIKV

**Fig 4:**
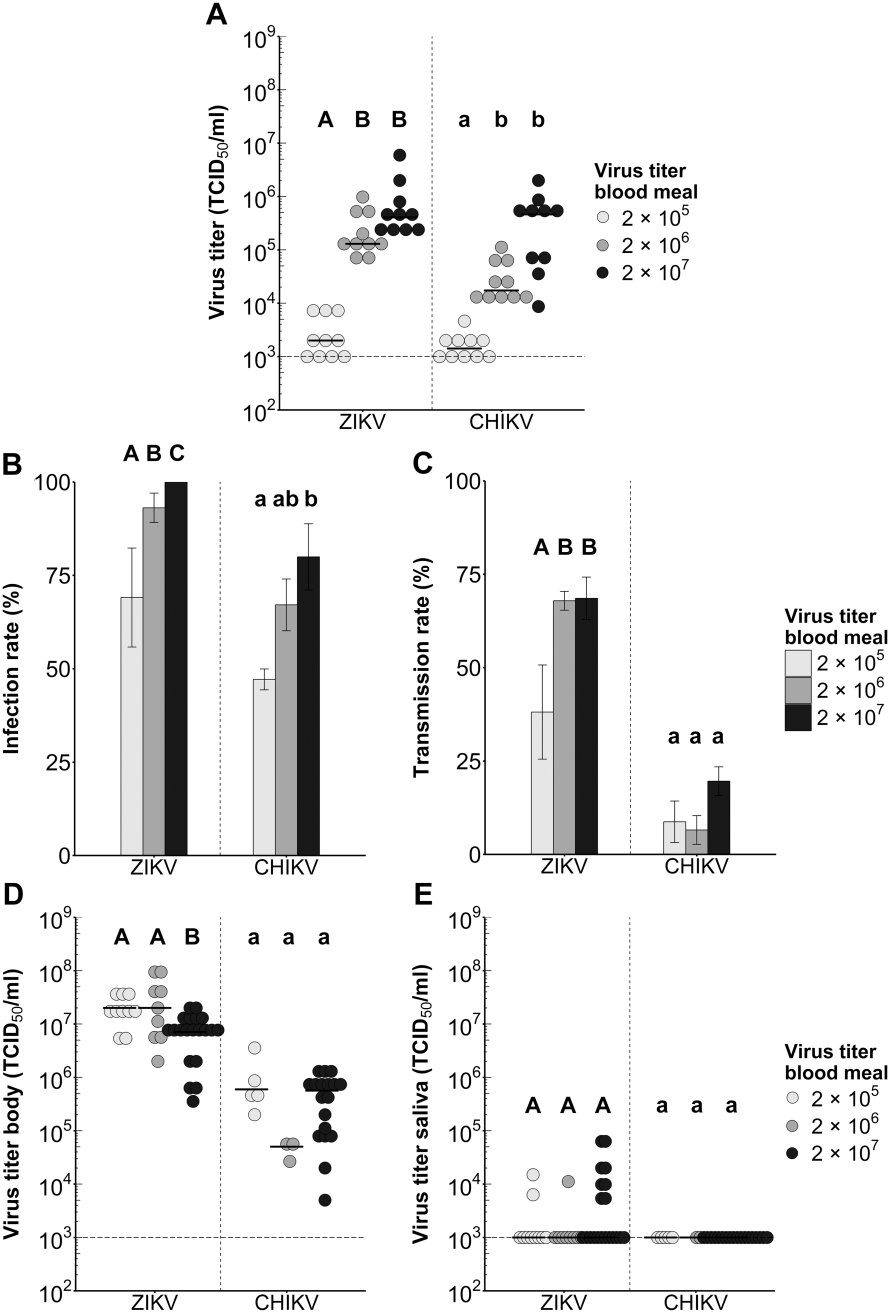
Infection and transmission rates of *Ae*. *aegypti* orally infected with different doses of ZIKV or CHIKV. Mosquitoes were inoculated with an infectious blood meal containing a dose of 2.0 × 10^5^, 2.0 × 10^6^, or 2.0 × 10^7^ TCID_50_/ml ZIKV^SUR^ or CHIKV^37997^. (A) Ingested virus titers of *Ae*. *aegypti* immediately after blood feeding. Each dot represents one mosquito body, and horizontal bars indicate median titers. The detection limit of the EPDA is indicated by the dashed line. Results were evaluated with a Kruskal-Wallis test. Significant differences between viral doses (*P* < 0.05) are indicated by different letters. (B) Infection and (C) transmission rates of *Ae*. *aegypti* mosquitoes at 14 dpi presented as the percentage of the total number of engorged mosquitoes. Shown are the mean percentages from three independent replicates. Error bars show the standard error of the mean. Sample size ranged between 48–101 female mosquitoes per treatment. Results were evaluated with Chi-squared test. Significant differences between viral doses (*P* < 0.05) are indicated by different letters. (D-E) Virus titers of CHIKV and ZIKV mosquito (D) bodies and (E) saliva. Each dot represents one mosquito body or saliva sample, and the horizontal bars indicate median titers. The detection limit of the EPDA is indicated by a dashed line. Results were evaluated with a Kruskal-Wallis test. Significant differences between viral doses (*P* < 0.05) are indicated by different letters.

The viral infectious dose in the blood meal is known to have a strong effect on the mosquito infection rates of mosquito-borne arboviruses [32,33]. Therefore, we determined the dose-dependent infection and transmission rates of ZIKV and CHIKV in *Ae*. *aegypti*. Female *Ae*. *aegypti* mosquitoes were offered an infectious blood meal containing 2.0 × 10^5^, 2.0 × 10^6^ or 2.0 × 10^7^ TCID_50_/ml of ZIKV or CHIKV. When comparing single- with co-infections it is important that the infectious blood meals contain equal virus titers and that the mosquitoes engorge similar numbers of infectious particles. To ensure that the infectious blood meals were completely homogenized and to validate our viral dilution series we froze a selection of engorged mosquito bodies directly after the blood meal and determined the virus titer by EPDA. Indeed, mosquitoes infected with increasing doses of infectious virus in the bloodmeal had increasing titers in their bodies. The ingested virus titers of mosquitoes that were infected with the lowest dose were significantly lower than those infected with the two highest doses (ZIKV: *P* < 0.01, CHIKV: *P* < 0.01; Fig 4A), although mosquitoes infected with the two highest doses were not significantly different amongst each other (*P* > 0.05).

The infection rates were determined at 14 dpi by infectivity assay of mosquito bodies and transmission rates by infectivity assay of saliva samples. Inoculation with 2.0 × 10^5^ TCID_50_/ml in the blood meal resulted in an infection rate of 65.3% for ZIKV (Fig 4B and Table 1). Increasing the infectious dose to 2.0 × 10^6^ or 2.0 × 10^7^ TCID_50_/ml significantly increased the ZIKV infection rate to 92.2% and 100% (*P* < 0.01). For CHIKV, inoculation with 2.0 × 10^5^, 2.0 × 10^6^ or 2.0 × 10^7^ TCID_50_/ml resulted in infection rates of 47.9%, 66.7%, or 81.2%, respectively. Inoculation with the highest CHIKV dose resulted in significantly higher infection rates compared to the lowest dose (*P* < 0.001). These results indicate that the mosquito infectious dose for *Ae*. *aegypti* is higher for CHIKV than ZIKV.

**Table 1:** Infection rates, transmission rates, and median titers of *Ae*. *aegypti* mosquitoes orally exposed to different doses of ZIKV and CHIKV. Infection and transmission rates determined at 14 dpi are presented as percentages (number of virus positive mosquito bodies or saliva samples / total number of engorged mosquitoes). Titers were determined for mosquitoes with a fully disseminated infection of ZIKV or CHIKV. The results represent the cumulative data from three independent biological replicates.

Virus	Dose (TCID_50_/ml)	Infection rate (%)	Transmission rate (%)	Median titer body (TCID_50_/ml)	Median titer saliva (TCID_50_/ml)
ZIKV^SUR^	2.0 × 10^5^	65.3 (32/49)	34.7 (17/49)	2.0 × 10^7^	1.0 × 10^3^
	2.0 × 10^6^	92.2 (47/51)	68.6 (35/51)	2.0 × 10^7^	1.0 × 10^3^
	2.0 × 10^7^	100 (101/101)	68.3 (69/101)	7.1 × 10^6^	1.0 × 10^3^
CHIKV^37997^	2.0 × 10^5^	47.9 (23/48)	10.4 (5/48)	6.0 × 10^5^	1.0 × 10^3^
	2.0 × 10^6^	66.7 (34/51)	5.9 (3/51)	5.0 × 10^4^	1.0 × 10^3^
	2.0 × 10^7^	81.2 (69/85)	21.2 (18/85)	5.7 × 10^5^	1.0 × 10^3^

In the same set of experiments, the mosquito saliva was collected by forced salivation assay and scored for the presence of virus to calculate the transmission rates. Transmission rates of 34.7% for ZIKV and 10.4% for CHIKV were reached with an infectious dose of 2.0 × 10^5^ TCID_50_/ml (Fig 4C and Table 1). With a viral titer in the blood meal of 2.0 × 10^6^ or 2.0 × 10^7^ TCID_50_/ml the transmission rates of ZIKV increased significantly to 68.6% and 68.3% (*P* < 0.01), whereas transmission rates for CHIKV of 5.9% and 21.2% were not significantly different as compared to the lowest dose (*P* > 0.05).

To observe whether increasing infectious doses in the blood meal lead to higher viral titers in the mosquito bodies and saliva we titrated both the mosquito bodies and saliva samples, of mosquitoes with a fully disseminated ZIKV or CHIKV infection (positive body and saliva) at 14 dpi. Median ZIKV titers in mosquito bodies reached 2.0 × 10^7^, 2.0 × 10^7^, and 7.1 × 10^6^ TCID_50_/ml for the respective inoculation doses of 2.0 × 10^5^, 2.0 × 10^6^, or 2.0 × 10^7^ TCID_50_/ml (Fig 4D). Median titers of mosquito bodies inoculated with 2.0 × 10^7^ of ZIKV were significantly lower compared to median titers of mosquito bodies inoculated with the lower doses (*P* < 0.05), indicating that a higher infectious dose in the blood meal does not necessary lead to a higher viral load in the mosquito. Median CHIKV titers were approximately 1–3 logs lower than ZIKV titers and reached values of 6.0 × 10^5^, 5.0 × 10^4^, and 5.7 × 10^5^ TCID_50_/ml for the respective inoculation doses of 2.0 × 10^5^, 2.0 × 10^6^, or 2.0 × 10^7^ (Fig 4D). No significant differences were found between the median titers of mosquito bodies inoculated with different doses of CHIKV (*P* > 0.05). In addition, median viral titers in virus-positive mosquito saliva samples were determined. All median viral titers of saliva samples were below the TCID_50_ detection limit, and no significant differences between saliva samples could be observed (*P* > 0.05; Fig 4E). These results show that *Ae*. *aegypti* is a competent vector for both ZIKV and CHIKV. Moreover, the relatively high mosquito infectious dose for CHIKV indicates that a higher viral dose in the blood meal should be used to study the effects of ZIKV and CHIKV co-infections.

### ZIKV and CHIKV are simultaneously transmitted by *Ae*. *aegypti* upon co-infection

**Fig 5:**
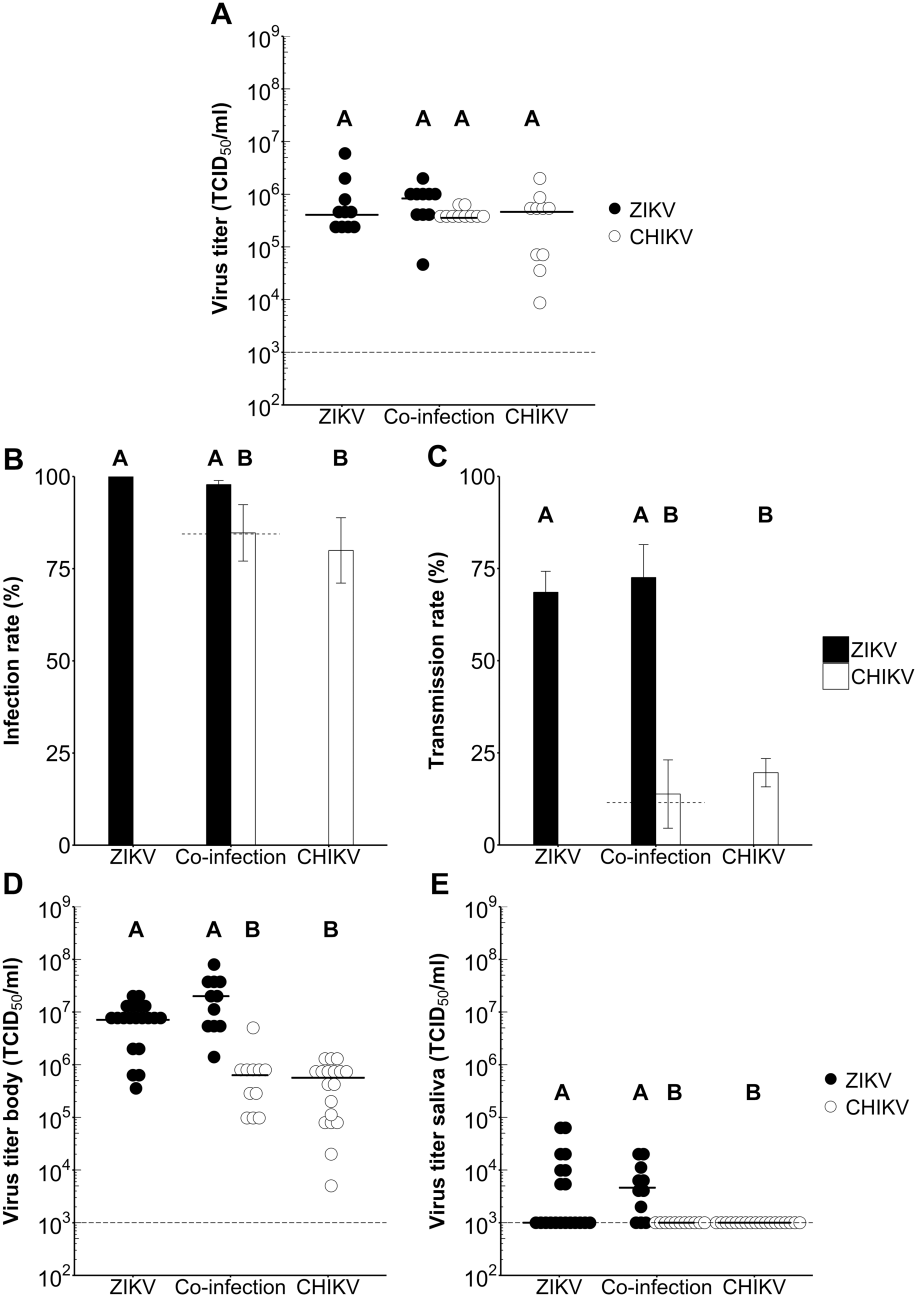
Infection and transmission rates of *Ae*. *aegypti* mosquitoes orally inoculated with ZIKV, CHIKV, or both viruses. Mosquitoes were orally infected with an infectious blood meal containing a dose of 2.0 × 10^7^ TCID_50_/ml ZIKV^SUR^, CHIKV^37997^, or both. (A) Ingested virus titers of *Ae*. *aegypti* immediately after blood feeding. Each dot represents one mosquito body, and the horizontal bars indicate median titers. The detection limit of the EPDA is indicated by a dashed line. Results were evaluated with a Kruskal-Wallis test. Significant differences between treatments (*P* < 0.05) are indicated by different letters. (B) Infection and (C) transmission rates of *Ae*. *aegypti* mosquitoes at 14 dpi presented as the percentage of the total number of engorged mosquitoes. The percentage of co-infected mosquitoes and saliva samples is indicated by the dashed line. Shown are the mean percentages from three independent replicates. Error bars show the standard error of the mean. Sample size ranged between 85–101 female mosquitoes per treatment. Results were evaluated with Chi-squared tests. Significant differences between treatments (*P* < 0.05) are indicated by different letters. (D-E) Virus titers of CHIKV and ZIKV mosquito (D) bodies and (E) saliva. Each dot represents one mosquito body or saliva sample, and the horizontal bars indicate median titers. The detection limit of the EPDA is indicated by a dashed line. Results were evaluated with a Kruskal-Wallis test. Significant differences between treatments (*P* < 0.05) are indicated by different letters.

Co-infections of arboviruses can affect their transmission potential by mosquito vectors and even exclude transmission of one virus [34,35]. To investigate the effect of co-infection on the infection and transmission of ZIKV and CHIKV, female *Ae*. *aegypti* mosquitoes were offered an infectious blood meal containing a dose of 2.0 × 10^7^ TCID_50_/ml of ZIKV, CHIKV, or both. Titrations of engorged mosquito bodies that were immediately frozen after the infectious blood meal, showed that the mosquitoes ingested equal amounts of CHIKV and ZIKV in the single- and co-infections (*P* = 0.24; Fig 5A). At 14 dpi, saliva was collected from the mosquitoes and the infection and transmission rates were determined by infectivity assay. ZIKV infection rates were 100% for the single-infection and 97.9% for the co-infection, which was not significantly different (*P* = 1.00; Fig 5B & Table 2). Similarly, no significant difference was found between infection rates of orally exposed mosquitoes to CHIKV in single- (81.2%) and co-infection with ZIKV (85.4%; *P* = 1.00). In both single- and co-infection, infection rates of mosquitoes orally exposed to CHIKV were significantly lower than those of mosquitoes exposed to ZIKV (*P* < 0.05). In total, 84.4% of mosquitoes that were simultaneously exposed to both viruses, were infected with both ZIKV and CHIKV. These results indicate that co-infection of ZIKV and CHIKV does not affect the infection rates in *Ae*. *aegypti*.

**Table 2:** Infection rates, transmission rates and median titers of *Ae*. *aegypti* mosquitoes orally exposed to ZIKV, CHIKV, or both ZIKV and CHIKV. Infection and transmission rates of mosquitoes in the co-infection treatment were determined as the percentage of mosquitoes with either ZIKV, CHIKV, or both viruses in their body or saliva, respectively, out of the total number of orally exposed mosquitoes within the respective treatment. Infection and transmission rates are presented as percentages (number of virus positive mosquito bodies or saliva samples / total number of engorged mosquitoes). Titers were determined for mosquitoes with a fully disseminated infection of ZIKV, CHIKV, or both. The results represent the cumulative data from three independent biological replicates.

Infection	Virus	Dose (TCID_50_/ml)	Infection rate (%)	Transmission rate (%)	Median titer body (TCID_50_/ml)	Median titer saliva (TCID_50_/ml)
Single-infection	ZIKV^SUR^	2.0 × 10^7^	100 (101/101)	68.3 (69/101)	7.1 × 10^6^	1.0 × 10^3^
	CHIKV^37997^	2.0 × 10^7^	81.2 (69/85)	21.2 (18/85)	5.7 × 10^5^	1.0 × 10^3^
Co-infection	ZIKV^SUR^	2.0 × 10^7^	97.9 (94/96)	72.9 (70/96)	2.0 × 10^7^	4.6 × 10^3^
	CHIKV^37997^	2.0 × 10^7^	85.4 (82/96)	14.6 (14/96)	6.3 × 10^5^	1.0 × 10^3^
	ZIKV^SUR^ & CHIKV^37997^		84.4 (81/96)	11.5 (11/96)		

Transmission rates of mosquitoes orally exposed to ZIKV were 68.3% for the single-infection and 72.9% for the co-infection, which was not significantly different (*P* = 1.00; Fig 5C & Table 2). For CHIKV, transmission rates were 21.2% for mosquitoes with a single-infection and 14.6% for mosquitoes with a co-infection, which was again not significantly different (*P* = 1.00). However, transmission rates of mosquitoes orally exposed to CHIKV were significantly lower than ZIKV exposed mosquitoes (*P* < 0.001). Importantly, 11.5% of mosquitoes that were simultaneously exposed to both viruses, had both ZIKV and CHIKV in their saliva, showing that *Ae*. *aegypti* can transmit both ZIKV and CHIKV via a single bite. In summary, these results show that simultaneous exposure can lead to concurrent transmission of both viruses without affecting the infection or transmission rates of ZIKV or CHIKV in *Ae*. *aegypti*.

Although no effect of co-infection on the infection and transmission rates was observed, there might be an effect on the viral titers in either the mosquito body or saliva that could have an effect on virus transmission. Therefore, viral titers were determined at 14 dpi for both mosquito bodies and saliva samples, of mosquitoes with fully disseminated infections of ZIKV, CHIKV, or both. ZIKV median titers in mosquito bodies reached 7.1 × 10^6^ after single- and 2.0 × 10^7^ TCID_50_/ml after co-infection, whereas CHIKV reached titers of 5.7 × 10^5^ after single- and 6.3 × 10^5^ TCID_50_/ml after co-infection (Fig 5D & Table 2). Median titers of both ZIKV and CHIKV in saliva samples reached 1.0–4.6 × 10^3^ TCID_50_/ml (Fig 5E & Table 2). Compared to single-infection, co-infection did not influence the titers of ZIKV or CHIKV in mosquito bodies or saliva (*P* >0.05). ZIKV titers were significantly higher than CHIKV titers in both mosquito bodies (*P* < 0.01) and saliva (*P* < 0.05; Fig 5D & 5E). These results show that co-infection with ZIKV and CHIKV does not affect the transmission potential of *Ae*. *aegypti* for either virus. Importantly, these experiments demonstrate for the first time that *Ae*. *aegypti* is intrinsically capable of transmitting ZIKV and CHIKV via a single bite.

### *Ae*. *aegypti* has a strong salivary gland barrier for CHIKV and a minor barrier for ZIKV

**Fig 6:**
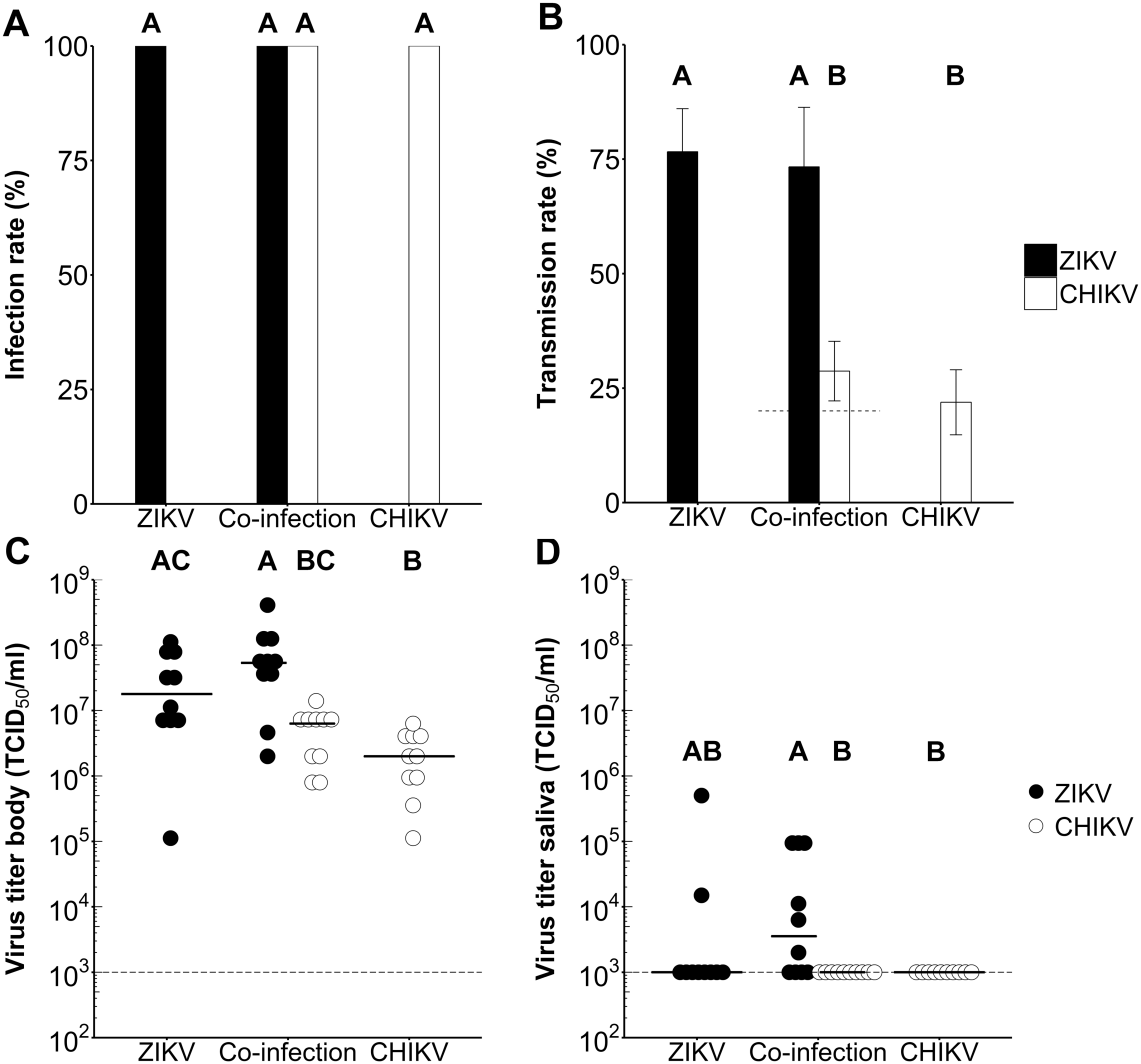
Infection and transmission rates of *Ae*. *aegypti* intrathoracically injected with ZIKV, CHIKV, or both viruses. Mosquitoes were infected through intrathoracic injections with a dose of 2.8 × 10^3^ TCID_50_ units of ZIKV^SUR^, CHIKV^37997^, or both. (A) Infection and (B) transmission rates of *Ae*. *aegypti* mosquitoes at 7 dpi presented as percentage of the total number of injected mosquitoes. The percentage of co-infected mosquito bodies and saliva samples is indicated by the dashed line. Shown are the mean percentages from three independent replicates. Error bars show the standard error of the mean. Sample size ranged between 48–49 female mosquitoes per treatment. Results were evaluated with Chi-squared tests, and corrected for multiple comparisons with the Bonferroni correction. Significant differences between treatments (*P* < 0.05) are indicated by different letters.(C-D). Virus titers of CHIKV and ZIKV mosquito (C) bodies and (D) saliva. Each dot represents one mosquito body, and the horizontal bars indicate median titers. The detection limit of the EPDA is indicated by a dashed line. Results were evaluated with a Kruskal-Wallis test, and Dunn’s test for multiple comparisons, corrected with the Bonferroni correction. Significant differences between treatments (*P* < 0.05) are indicated by different letters.

We observed high infection rates for ZIKV and CHIKV, but the transmission rates for CHIKV were notably lower as compared to ZIKV. This substantial difference in transmissibility of ZIKV and CHIKV by *Ae*. *aegypti* could be due to the presence of a midgut escape barrier, a salivary gland barrier or both. To discriminate between these two possibilities, female *Ae*. *aegypti* mosquitoes were intrathoracically injected (to by-pass the midgut barriers) with 2.8 × 10^3^ TCID_50_ units of ZIKV, CHIKV or both viruses. After 7 days, mosquito saliva was collected and bodies and saliva were tested for presence of virus by infectivity assay. Injection with ZIKV, CHIKV, and both viruses resulted in all cases in 100% infection rates (Fig 6A & Table 3). Transmission rates of mosquitoes injected with ZIKV were similar for the single-infection (77.6%) and after co-infection (68.8%; *P* = 1.00; Fig 6B & Table 3). For CHIKV the transmission rates were also similar for the single-infection (22.9%) and the co-infection (27.1%; *P* = 1.00), again suggesting that ZIKV and CHIKV do not interfere. Transmission rates of mosquitoes intrathoracically injected with CHIKV were significantly lower compared to ZIKV (*P* < 0.001). In total, 20.8% of the mosquitoes that were simultaneously exposed to both viruses had both ZIKV and CHIKV in their saliva.

**Table 3:** Infection rates, transmission rates and median titers of *Ae*. *aegypti* mosquitoes intrathoracically injected with ZIKV, CHIKV, or both ZIKV and CHIKV. Infection and transmission rates of mosquitoes were determined as the percentage of mosquitoes with either ZIKV, CHIKV, or both viruses in their body or saliva, respectively, out of the total number of injected mosquitoes within the respective treatment. Infection and transmission rates are presented as percentages (number of virus positive mosquito bodies or saliva samples / total number of engorged mosquitoes). Titers were determined for mosquitoes with a fully disseminated infection of ZIKV, CHIKV, or both. The results represent the cumulative data from three independent biological replicates.

Infection	Virus	Dose (TCID_50_/ml)	Infection rate (%)	Transmission rate (%)	Median titer body (TCID_50_/ml)	Median titer saliva (TCID_50_/ml)
Single-infection	ZIKV^SUR^	2.8 × 10^3^	100 (49/49)	77.6 (38/49)	2.0 × 10^7^	1.0 × 10^3^
	CHIKV^37997^	2.8 × 10^3^	100 (48/48)	22.9 (11/48)	2.0 × 10^6^	1.0 × 10^3^
Co-infection	ZIKV^SUR^	2.8 × 10^3^	100 (48/48)	68.8 (33/48)	5.4 × 10^7^	4.2 × 10^3^
	CHIKV^37997^	2.8 × 10^3^	100 (48/48)	27.1 (13/48)	6.3 × 10^6^	1.0 × 10^3^
	ZIKV^SUR^ & CHIKV^37997^		100 (48/48)	20.8 (10/48)		

Viral titers were again determined for mosquito bodies and saliva samples of mosquitoes with a fully disseminated infection, which were injected with ZIKV, CHIKV, or both viruses simultaneously. ZIKV reached mosquito body titers of 2.0 × 10^7^ TCID_50_/ml after single- and 5.4 × 10^7^ TCID_50_/ml after co-infections, whereas CHIKV reached titers of 2.0 × 10^6^ after single- and 6.3 × 10^6^ TCID_50_/ml after co-infection (Fig 6C & Table 3). Median titers of both ZIKV and CHIKV in saliva samples reached 1.0–4.2 × 10^3^ TCID_50_/ml (Fig 6D & Table 3). Compared to single-infection, co-infection did not influence the titers of ZIKV or CHIKV in mosquito bodies and saliva (*P* > 0.05; Fig 6C & 6D).

The low transmission rates of CHIKV as compared to the high infection rates after both blood meal infection and intrathoracic injection (Figs 5C & 6B), indicate the presence of a salivary gland barrier that prevents the virus from dissemination into the saliva. For ZIKV, the transmission rates after intrathoracic injections and blood meal infections are only slightly lower than the infection rates. This suggests that for ZIKV the salivary glands form a minor barrier for accumulation of infectious virus in the saliva.

## Discussion

Since the start of the global spread of ZIKV, this virus co-circulates with CHIKV in many parts of the world. There is an increase in the number of reports describing co-infections of ZIKV and CHIKV (and also DENV) in human patients, but the extent of co-infection in field-collected mosquitoes is not clear. The aim of this study was to assess whether the predominant vector of ZIKV and CHIKV in the Americas, *Ae*. *aegypti*, is able to transmit both viruses simultaneously, and whether co-infection may change the vector competence for either virus. Here we show that *Ae*. *aegypti* mosquitoes can indeed simultaneously transmit ZIKV and CHIKV via a single bite. Infection with both ZIKV and CHIKV did not result in lowered infection or transmission rates for either virus, although *Ae*. *aegypti* was shown to be a more efficient vector for ZIKV as compared to CHIKV. Finally, we show that *Ae*. *aegypti* mosquitoes have a salivary gland barrier for both CHIKV and ZIKV.

Triple co-infections with ZIKV, CHIKV and DENV have already been reported in patients from Colombia and Nicaragua [21–23] and several cases of ZIKV and CHIKV co-infections in patients have been reported [18–20]. These observations indicate that a single mosquito could take a blood meal containing multiple arboviruses, potentially resulting in the transmission of different viruses simultaneously. Simultaneous transmission of alphaviruses and flaviviruses by *Ae*. *albopictus* has been reported for DENV and CHIKV [36], and for DENV and Sindbis virus [34]. In another study, CHIKV and DENV co-transmission by either *Ae*. *aegypti* or *Ae*. *albopictus* only occurred after sequential blood meals, but not after simultaneous infection in a single blood meal [37]. Furthermore, co-infection of Sindbis virus and DENV greatly decreases both the infection and transmission rates of both viruses [34]. In contrast to these studies, our results clearly show that ZIKV and CHIKV do not interfere with each other in either their infection or transmission by *Ae*. *aegypti*.

Our results show that ZIKV and CHIKV can replicate simultaneously in a single cell of both the mammalian host and mosquito vector. Furthermore, we show that ZIKV and CHIKV can simultaneously disseminate to the saliva of *Ae*. *aegypti* mosquitoes, indicating that co-infections do not strongly interfere with virus replication. The effect of co-infection of arboviruses on virus replication is still poorly understood. One explanation for interference between different viruses is superinfection exclusion, where infection of a primary virus excludes secondary infection with the same or a different virus [30]. The primary virus infection could induce the host-immune response, claim important cellular factors required for viral replication, or produce defective interfering particles, that suppress replication of a secondary viral infection. Alternatively, primary infection may lead to suppression of the mosquito’s antiviral responses, leading to enhanced infection of a secondary virus (reviewed in [30]). Potentially, asynchronous co-infections of CHIKV and ZIKV in *Ae*. *aegypti* mosquitoes could result in detectable interference and may affect the infection and transmission rates.

However, viral interference was observed in growth curves in Vero cells with co-infection resulting in decreased ZIKV titers. The interference in Vero cells is likely due to a decrease in cell viability as a result of CHIKV infection, leading to high cell death and, thus, less ZIKV production. In contrast to what we observed in mammalian cells, virus replication and cell viability were not affected when C6/36 or Aag2 cells were infected or co-infected by both viruses. The absence of detectable interference between the two viruses on virus replication in mosquito cell lines supports our findings on vector competence of *Ae*. *aegypti* mosquitoes infected with both viruses. CHIKV replicates in spherules at the plasma membrane [38] whereas flaviviruses replicate in perinuclear regions of the endoplasmic reticulum [39], which may explain the lack of interference between both viruses. Furthermore, tampering of virus replication by defective interfering particles, which often contributes to viral interference, is less problematic with alpha- and flavivirus co-infections [40]. Potentially, co-infections of multiple flaviviruses (e.g. DENV and ZIKV) may have a stronger effect on vector competence. Our results support the role of *Ae*. *aegypti* as a competent vector for ZIKV and CHIKV.

Previous vector competence studies reported infection rates between 70–100% in *Ae*. *aegypti* for ZIKV [6,9,41].

Infection rates reported here for the ZIKV^SUR^ strain are in line with these findings with 100% infection after administering a high dose of 2.0 × 10^7^ blood meal, and 65–90% with a ten- to hundred-fold lower dose of ZIKV in the blood meal. The transmission rates of 35–70% for the ZIKV^SUR^ strain are higher than some previously reported transmission rates, which ranged between 10–30% for the American strains of ZIKV in Brazilian and Mexican *Ae*. *aegypti* mosquitoes [9,41]. However, studies with Australian and Poza Rica strain *Ae*. *aegypti* reported transmission rates between 70–85% [7,41], which is more in the range of our findings. For the CHIKV^37997^ strain, the infection rates reported here (50–80%) are higher than some previous reports on CHIKV vector competence, which range between 10–30% [42,43]. However, another comprehensive study that investigated the vector competence of ten different *Ae*. *aegypti* populations for CHIKV reported high infection and dissemination rates between 90–100% [15]. Transmission rates of CHIKV by *Ae*. *aegypti* mostly range between 40–60% with exceptions to some virus-vector strain combinations that report low transmission rates [8,15,43]. These findings confirm that vector competence is highly variable and dependent on the specific combination of viral strain and mosquito population.

In order for an arbovirus to accumulate in the saliva it has to pass the midgut infection- and escape barriers and the salivary gland infection- and escape barriers [44]. A midgut barrier for CHIKV has previously been reported in *Ae*. *aegypti* [45] and *Ae*. *albopictus* [46]. Here, we report infection rates of up to 80% for CHIKV, suggesting that the midgut does not form a strong barrier against CHIKV infection in our *Ae*. *aegypti* colony. However, CHIKV transmission rates of maximum 20% after an infectious blood meal or intrathoracic injections indicate the presence of a mosquito salivary gland barrier. The presence of a salivary gland barrier is indicated by a low percentage of mosquitoes with a salivary gland infection when a larger percentage of mosquitoes reaches a disseminated infection. We determined transmission rates of CHIKV of maximum 21% after oral exposure while an 81% infection rate was observed, suggesting that the mosquito colony used has a strong salivary gland barrier to the CHIKV^37997^ strain. Additionally virus-positive saliva samples had a low viral titer for both ZIKV and CHIKV. Although we did not quantify the amount of saliva that the mosquitoes excreted, this suggests that there is indeed a salivary gland barrier for both viruses that prevents the accumulation of high viral titers in the saliva. We determined that the transmission rates after intrathoracic injections were similar to the transmission rates after an infectious blood meal at 7 dpi. Potentially CHIKV and ZIKV may require longer incubation periods between 7 and 14 days to successfully infect the salivary glands and reach the saliva. However, a salivary gland escape barrier has earlier been described for the CHIKV^37997^ strain, where only 60% of the infected mosquitoes had virus-positive saliva at 7 dpi [47].

It is surprising that ZIKV spread so rapidly from its original, natural range to territories in the Pacific (Micronesia, Eastern Island, French Polynesia), and subsequently to South- and North-America between 2007 and 2015 [2]. Several hypotheses have been proposed for the rapid dissemination of ZIKV throughout the Americas. First, genetic changes of ZIKV strains could result in adaptations that make the strain that circulates in the Americas more virulent. Evidence for this comes from a comparative genomic study that indicated 15 amino acid substitutions in epidemic strains compared to pre-epidemic strains [48]. However, an African ZIKV isolate was shown to outcompete the American strain *in vitro* and *in vivo* suggesting that transmission of the epidemic strains is not enhanced [49]. Secondly, the presence of a large naïve and susceptible human population in combination with high densities of anthropophilic mosquitoes might have accelerated the spread of ZIKV. And thirdly, co-infection of mosquitoes with ZIKV and other arboviruses such as CHIKV and DENV or both may have a positive effect on the vector competence of mosquitoes, resulting in increased transmission rates and faster spread of the viruses. We now show for the first time that mosquito co-infections of ZIKV and CHIKV can indeed occur, without altering the vector competence of *Ae*. *aegypti* for either virus. Importantly, our results suggest that patients reported with ZIKV and CHIKV co-infections could have been infected with both viruses via the bite of a single *Ae*. *aegypti* mosquito. However, the proportion of co-infected mosquitoes in a population of *Ae*. *aegypti* mosquitoes is expected to be extremely small. We therefore consider it unlikely that co-infections of multiple arboviruses contribute to the rapid dissemination of ZIKV across the Americas.

In summary, this study shows that *Ae*. *aegypti* can transmit ZIKV and CHIKV simultaneously by a single mosquito bite. Additionally, we show that *Ae*. *aegypti* has a higher vector competence for ZIKV than for CHIKV and that co-infections do not affect the vector competence. By comparison of infections via the blood meal with intrathoracic injections we show that *Ae*. *aegypti* has a strong salivary gland barrier for CHIKV and a minor salivary gland barrier for ZIKV. Finally, studies with cell lines show that ZIKV virus production is decreased in mammalian, but not mosquito cells, due to induction of cytopathicity by CHIKV. The outcomes of this research provide novel insights into the effects of co-infections on the transmission of arboviruses by mosquitoes.
